# Effects of iron homeostasis on epigenetic age acceleration: a two-sample Mendelian randomization study

**DOI:** 10.1186/s13148-023-01575-w

**Published:** 2023-10-07

**Authors:** Zhihao Wang, Yi Liu, Shuxin Zhang, Yunbo Yuan, Siliang Chen, Wenhao Li, Mingrong Zuo, Yufan Xiang, Tengfei Li, Wanchun Yang, Yuan Yang, Yanhui Liu

**Affiliations:** 1 Department of Neurosurgery, West China Hospital, Sichuan University, Chengdu, China; 2 Department of Thoracic Surgery and Institute of Thoracic Oncology, West China Hospital, Sichuan University, Chengdu, China; 3 Department of Critical Care Medicine, West China Hospital, Sichuan University, Chengdu, China

**Keywords:** Iron metabolism, Iron homeostasis, Senescence, Aging, Epigenetic age acceleration, Mendelian randomization

## Abstract

**Background:**

Epigenetic clocks constructed from DNA methylation patterns have emerged as excellent predictors of aging and aging-related health outcomes. Iron, a crucial element, is meticulously regulated within organisms, a phenomenon referred as iron homeostasis. Previous researches have demonstrated the sophisticated connection between aging and iron homeostasis. However, their causal relationship remains relatively unexplored.

**Results:**

Through two-sample Mendelian randomization (MR) utilizing the random effect inverse variance weighted (IVW) method, each standard deviation (SD) increase in serum iron was associated with increased GrimAge acceleration (GrimAA, Beta_IVW_ = 0.27, P = 8.54E−03 in 2014 datasets; Beta_IVW_ = 0.31, P = 1.25E−02 in 2021 datasets), HannumAge acceleration (HannumAA, Beta_IVW_ = 0.32, P = 4.50E−03 in 2014 datasets; Beta_IVW_ = 0.32, P = 8.03E−03 in 2021 datasets) and Intrinsic epigenetic age acceleration (IEAA, Beta_IVW_ = 0.34, P = 5.33E−04 in 2014 datasets; Beta_IVW_ = 0.49, P = 9.94E−04 in 2021 datasets). Similar results were also observed in transferrin saturation. While transferrin manifested a negative association with epigenetic age accelerations (EAAs) sensitivity analyses. Besides, lack of solid evidence to support a causal relationship from EAAs to iron-related biomarkers.

**Conclusions:**

The results of present investigation unveiled the causality of iron overload on acceleration of epigenetic clocks. Researches are warranted to illuminate the underlying mechanisms and formulate strategies for potential interventions.

**Supplementary Information:**

The online version contains supplementary material available at 10.1186/s13148-023-01575-w.

## Introduction

Aging is a natural process of all organisms characterized by loss of physiological integrity, function decline and vulnerable to death in a time-dependent manner [1]. Iron is one of the most essential transition metals in human body [2]. The balance of iron metabolism, also known as iron homeostasis, is strictly regulated due to its crucial role in erythropoiesis, oxidative phosphorylation and redox reaction [3]. Evidences have connected altered iron homeostasis with biological aging. For example, epidemiological research reported that over 10% of both men and women aged 65 years or older were anemic in the US, in which iron deficiency made up approximately 20% of all anemia cases [4]. Chronic inflammation of the elder people might also contribute to the alteration of serum iron biomarkers, causing iron deficiency and impaired iron mobilization [3, 5]. On the other hand, cellular iron accumulation in older individuals was observed. Serum level of ferritin, which reflected the storage of iron, was reported to be increasing with age and negatively associated with telomere length [6, 7]. Iron overload in cell induced the accumulation of lipofuscin, which was considered one of the hallmarks of aging and could be cytotoxic [5, 8]. However, little was known about the change of total iron content of body with age, as well as the relationship between iron homeostasis and aging.

The lifespan of organism is programed by its genetic information and influenced by its environment [9]. Distinct from conservative DNA sequence, the epigenome regulated gene expression based on a range of chemical modifications that were reversible [10]. Epigenetic alteration was proposed as one of the twelve hallmarks of aging and participated in the pathogenesis of several age-related diseases [1]. At present, the best characterized mark of epigenome is DNA methylation, a methyl group added on the fifth carbon of a cytosine residue, which could also be influenced by environmental factors, including the passage of time [11]. Thus, the epigenetic clocks, based on the DNA methylation status of human and their chronological age and health-related outcomes, were built to discover the impact of both genetic and environmental factors on human aging [12]. Epigenetic age acceleration (EAA) was used to describe individuals with greater epigenetic-clock-estimated age than their true chronological age, indicating worse health outcome [13]. Although iron homeostasis was in connection with aging, no research regarding the relationship between epigenetic clocks or EAA and iron homeostasis has been conducted.

Utilizing outcomes from genome-wide association studies (GWAS), Mendelian randomization (MR) has been widely used in discovering causality between exposure factors and outcomes and has a better performance in controlling confounding and reverse causation [14]. To achieve MR analysis, single nucleotide polymorphisms (SNPs) are selected as instrumental variables (IVs) with three rules, IVs must be (1) associated with the exposure; (2) independent of all confounders of the exposure–outcome association; and (3) independent of the outcome [15]. McCartney et al. conducted a GWAS of four epigenetic clocks, and subsequent MR analysis identified several risk factors of EAAs [16]. Based on the GWAS statistics, Pan et al. have reported bidirectional causal relationships between EAAs and kidney function [17]. To our knowledge, no MR analysis has been conducted exploring the causal relationship between iron homeostasis and EAAs.

In this study, we conducted a two-sample MR analyses with summarized GWAS data mentioned above to investigate the causal relationship between iron homeostasis and EAAs. The results might provide new evidence for further research on intervening iron homeostasis of the elderly and its potential mechanics.

## Results

### Genetic instruments selection

Selection of qualified SNPs from summarized GWAS data was conducted, and the number of SNPs in every process is presented in Additional file 1: Figure S1. Notably, lack of qualified SNPs from pancreas iron content, thus 183 of SNPs that met the threshold of 5E−06 were elevated. Total F-statistics of SNPs were all larger than 10, indicating strong IVs.

### Causal effects of plasmatic iron biomarkers on EAAs

**Fig. 1:**
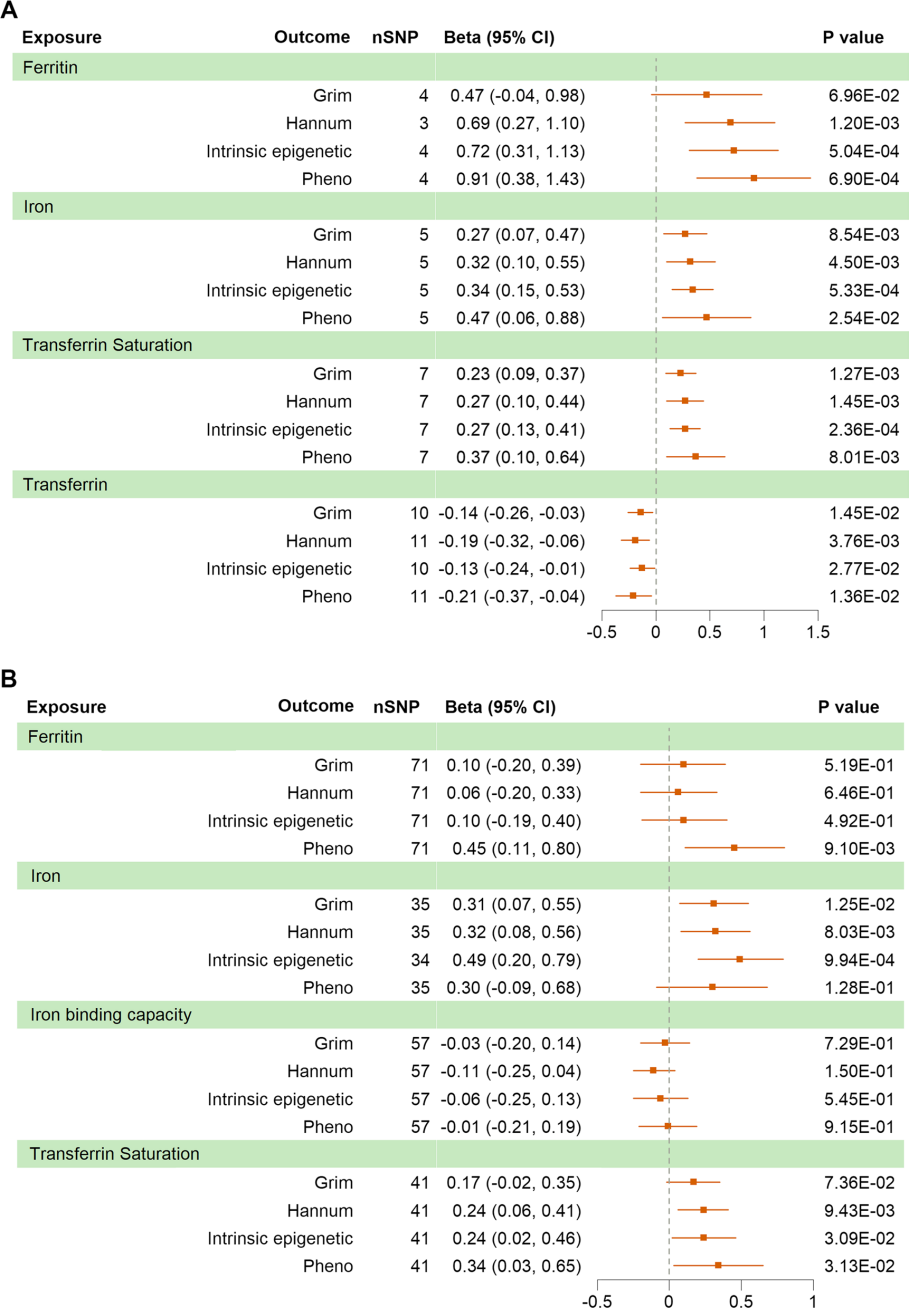
MR analyses of 2014 (**A**) and 2021 (**B**) datasets with epigenetic aging accelerations. MR, Mendelian randomization; SNP, single nucleotide polymorphism

Random effect inverse variance weighted (IVW) analyses were carried out with iron-related traits from three meta-GWAS as exposures and EAAs as outcomes. Serum iron, ferritin and transferrin saturation were simultaneously obtained from two independent GWAS (namely 2014 datasets and 2021 datasets based on the year of publish). As plotted in Fig. 1A, B, genetically predicted serum iron was significantly associated with genetic predisposition to GrimAge acceleration (GrimAA, Beta_IVW_ = 0.27 years per standard deviation (SD) increase in serum iron, P = 8.54E−03 in 2014 datasets; Beta_IVW_ = 0.31, P = 1.25E−02 in 2021 datasets), HannumAge acceleration (HannumAA, Beta_IVW_ = 0.32, P = 4.50E−03 in 2014 datasets; Beta_IVW_ = 0.32, P = 8.03E−03 in 2021 datasets) and intrinsic epigenetic age acceleration (IEAA, Beta_IVW_ = 0.34, P = 5.33E−04 in 2014 datasets; Beta_IVW_ = 0.49, P = 9.94E−04 in 2021 datasets), but partially significant in PhenoAge acceleration (PhenoAA, Beta_IVW_ = 0.47, P = 2.54E−02 in 2014 datasets; Beta_IVW_ = 0.30, P = 1.28E−01 in 2021 datasets). Transferrin saturation of both datasets demonstrated significant association with HannumAA (Beta_IVW_ = 0.27 years per SD increase in transferrin saturation, P = 1.45E−03 in 2014 datasets; Beta_IVW_ = 0.24, P = 9.43E−03 in 2021 datasets), IEAA (Beta_IVW_ = 0.27, P = 2.36E−04 in 2014 datasets; Beta_IVW_ = 0.24, P = 3.09E−02 in 2021 datasets) and PhenoAA (Beta_IVW_ = 0.37, P = 8.01E−03 in 2014 datasets; Beta_IVW_ = 0.34, P = 3.13E−02 in 2021 datasets), but lacked significance with GrimAA in 2021 datasets (Beta_IVW_ = 0.23, P = 1.27E−03 in 2014 datasets; Beta_IVW_ = 0.17, P = 7.36E−02 in 2021 datasets). As for ferritin, significant results were observed in HannumAA (Beta_IVW_ = 0.69 years per SD increase in ferritin, P = 1.20E−03), IEAA (Beta_IVW_ = 0.72, P = 5.04E−04) and PhenoAA (Beta_IVW_ = 0.91, P = 6.90E−04) from 2014 datasets, as well as PhenoAA (Beta_IVW_ = 0.45, P = 9.10E−03) from 2021 datasets. The same negative direction of associations was obtained in genetically predicted transferrin from 2014 datasets and genetically predicted total iron binding capacity (TIBC) from 2021 datasets, the former was significant with GrimAA (Beta_IVW_ = -0.14 years per SD increase in transferrin, P = 1.45E−02), HannumAA (Beta_IVW_ = -0.19, P = 3.76E−03), IEAA (Beta_IVW_ = −0.13, P = 2.77E−02) and PhenoAA (Beta_IVW_ = − 0.21, P = 1.36E−02), while the latter was not.

In sensitivity analyses, opposite directions were found in part of analyses of ferritin and TIBC from 2021 datasets. Notably, severe heterogeneity was detected in serum iron from 2014 datasets to PhenoAA (I2 = 66.5%). No pleiotropy was observed among all analyses. Results remained consistent after adjusting outliners in serum iron and transferrin saturation from 2021 datasets. Taken together, serum iron might be the potential cause of acceleration of GrimAge, HannumAge and intrinsic epigenetic age. Transferrin saturation was significantly associated with increasing of GrimAA, HannumAA, IEAA and PhenoAA. Ferritin was associated with elevated PhenoAA. All the above results were significant in two datasets and validated in sensitivity analyses. Serum transferrin demonstrated negative association with the four EAAs, but results of TIBC were only significant in IEAA and PhenoAA. Complete results are exhibited in Tables 1 and 2.

**Table 1:** Sensitivity analyses of serum iron biomarkers in 2014 datasets with epigenetic aging accelerations

Exposure	Outcome	Weighted median		MR-egger regression		Heterogeneity^a^	MR-PRESSO outlier detect^b^		Pleiotropy^c^
		Beta (95% CI)	P value	Beta (95% CI)	P value		Beta (95% CI)	P Value	
Ferritin	Grim	0.76 (0.23, 1.29)	4.82E− 03	0.76 (− 0.33, 1.85)	3.04E−01	I2 = 39.6%; Cochrane Q = 5; P = 0.174	No significant outliers		Intercept = -0.031; P = 0.603
Ferritin	Hannum	0.82 (0.33, 1.31)	1.04E−03	0.83 (− 0.25, 1.91)	3.74E−01	I2 = 0%; Cochrane Q = 2; P = 0.380	No significant outliers		Intercept = -0.018; P = 0.812
Ferritin	IE	0.75 (0.27, 1.24)	2.29E−03	0.98 (0.14, 1.82)	1.51E−01	I2 = 1.6%; Cochrane Q = 3; P = 0.384	No significant outliers		Intercept = -0.028; P = 0.554
Ferritin	Pheno	0.80 (0.18, 1.41)	1.18E−02	0.77 (− 0.43, 1.97)	3.34E−01	I2 = 6.7%; Cochrane Q = 3; P = 0.360	No significant outliers		Intercept = 0.014; P = 0.820
Iron	Grim	0.30 (0.06, 0.55)	1.33E−02	0.49 (0.09, 0.90)	9.77E−02	I2 = 14.5%; Cochrane Q = 5; P = 0.322	No significant outliers		Intercept = -0.045; P = 0.309
Iron	Hannum	0.31 (0.08, 0.53)	8.00E−03	0.54 (0.06, 1.01)	1.15E−01	I2 = 32.1%; Cochrane Q = 6; P = 0.208	No significant outliers		Intercept = -0.043; P = 0.396
Iron	IE	0.27 (0.02, 0.51)	3.16E−02	0.65 (0.25, 1.06)	5.09E−02	I2 = 0%; Cochrane Q = 3; P = 0.480	No significant outliers		Intercept = -0.064; P = 0.180
Iron	Pheno	0.55 (0.24, 0.85)	4.81E−04	0.60 (− 0.40, 1.6)	3.24E−01	I2 = 66.5%; Cochrane Q = 12; P = 0.018	No significant outliers		Intercept = -0.027; P = 0.787
Transferrin saturation	Grim	0.27 (0.10, 0.44)	1.62E−03	0.28 (0.03, 0.52)	7.80E−02	I2 = 0%; Cochrane Q = 3; P = 0.856	No significant outliers		Intercept = -0.011; P = 0.678
Transferrin saturation	Hannum	0.28 (0.12, 0.44)	7.46E−04	0.32 (0.01, 0.63)	9.98E−02	I2 = 30.3%; Cochrane Q = 9; P = 0.196	No significant outliers		Intercept = -0.013; P = 0.716
Transferrin saturation	IE	0.28 (0.11, 0.44)	9.04E−04	0.41 (0.16, 0.66)	2.36E−02	I2 = 0%; Cochrane Q = 2; P = 0.870	No significant outliers		Intercept = -0.035; P = 0.238
Transferrin saturation	Pheno	0.36 (0.14, 0.58)	1.49E−03	0.33 (− 0.19, 0.85)	2.67E−01	I2 = 56.8%; Cochrane Q = 14; P = 0.031	No significant outliers		Intercept = 0.010; P = 0.869
Transferrin	Grim	− 0.05 (− 0.21, 0.12)	5.68E−01	− 0.18 (− 0.35, 0.01)	8.77E−02	I2 = 4%; Cochrane Q = 9; P = 0.403	No significant outliers		Intercept = 0.009; P = 0.633
Transferrin	Hannum	− 0.12 (− 0.29, 0.05)	1.79E−01	− 0.15 (− 0.34, 0.05)	1.82E−01	I2 = 29.5%; Cochrane Q = 14; P = 0.164	No significant outliers		Intercept = -0.012; P = 0.530
Transferrin	IE	− 0.03 (− 0.19, 0.13)	6.71E−01	− 0.17 (− 0.34, 0.02)	9.29E−02	I2 = 0.7%; Cochrane Q = 9; P = 0.432	No significant outliers		Intercept = 0.011; P = 0.547
Transferrin	Pheno	− 0.17 (− 0.36, 0.03)	9.24E−02	− 0.19 (− 0.44, 0.07)	1.83E−01	I2 = 27.4%; Cochrane Q = 14; P = 0.184	No significant outliers		Intercept = -0.005; P = 0.830

^a^Heterogeneity in the random effect IVW methods was reported

^b^MR-PRESSO (NbDistribution = 10,000, P < 0.05)

^c^MR-Egger was used to detect Pleiotropy. There is no pleiotropy was observed among all analyses (P > 0.05)

CI, confidence interval, MR-PRESSO, Mendelian Randomization Pleiotropy RESidual Sum and Outlier, IE Intrinsic epigenetic

**Table 2:** Sensitivity analyses of serum iron biomarkers in 2021 datasets with epigenetic aging accelerations

Exposure	Outcome	Weighted median		MR-egger regression		Heterogeneity^a^	MR-PRESSO outlier detect^b^		Pleiotropy^c^
		Beta (95% CI)	P Value	Beta (95% CI)	P value		Beta (95% CI)	P Value	
Ferritin	Grim	− 0.17 (− 0.57, 0.22)	3.86E−01	0.33 (− 0.26, 0.92)	2.77E−01	I2 = 30.9%; Cochrane Q = 101; P = 0.008	No significant outliers		Intercept = -0.010; P = 0.374
Ferritin	Hannum	− 0.08 (− 0.45, 0.30)	6.85E−01	0.35 (− 0.17, 0.87)	1.95E−01	I2 = 17%; Cochrane Q = 84; P = 0.116	No significant outliers		Intercept = -0.012; P = 0.217
Ferritin	IE	0.07 (− 0.35, 0.49)	7.53E−01	0.16 (− 0.43, 0.76)	5.91E−01	I2 = 30.7%; Cochrane Q = 101; P = 0.009	-0.02 (-0.31, 0.27)	8.88E−01	Intercept = -0.003; P = 0.821
Ferritin	Pheno	0.24 (− 0.27, 0.75)	3.58E−01	0.70 (0.02, 1.39)	4.90E−02	I2 = 17%; Cochrane Q = 84; P = 0.117	No significant outliers		Intercept = -0.011; P = 0.418
Iron	Grim	0.53 (0.18, 0.87)	2.82E−03	0.45 (0.06, 0.84)	3.01E−02	I2 = 0%; Cochrane Q = 28; P = 0.743	No significant outliers		Intercept = -0.011; P = 0.374
Iron	Hannum	0.47 (0.12, 0.81)	7.57E−03	0.55 (0.18, 0.93)	6.71E−03	I2 = 0%; Cochrane Q = 28; P = 0.75	No significant outliers		Intercept = -0.019; P = 0.125
Iron	IE	0.67 (0.29, 1.04)	5.01E−04	0.75 (0.30, 1.21)	2.79E−03	I2 = 29.5%; Cochrane Q = 47; P = 0.056	No significant outliers		Intercept = -0.021; P = 0.159
Iron	Pheno	0.24 (− 0.21, 0.68)	2.93E−01	0.45 (− 0.16, 1.07)	1.60E−01	I2 = 35.8%; Cochrane Q = 53; P = 0.02	0.36 (0.03, 0.69)	3.96E−02	Intercept = -0.012; P = 0.534
Iron binding capacity	Grim	− 0.09 (− 0.32, 0.15)	4.71E−01	0.05 (− 0.25, 0.34)	7.66E−01	I2 = 27.8%; Cochrane Q = 78; P = 0.03	No significant outliers		Intercept = -0.007; P = 0.553
Iron binding capacity	Hannum	0.01 (− 0.20, 0.22)	9.18E−01	− 0.11 (− 0.37, 0.14)	3.94E−01	I2 = 6.6%; Cochrane Q = 60; P = 0.333	No significant outliers		Intercept = 0.001; P = 0.951
Iron binding capacity	IE	− 0.02 (− 0.28, 0.23)	8.58E−01	− 0.15 (− 0.49, 0.18)	3.72E−01	I2 = 42.3%; Cochrane Q = 97; P = 0.001	No significant outliers		Intercept = 0.009; P = 0.501
Iron binding capacity	Pheno	− 0.17 (− 0.44, 0.10)	2.19E−01	− 0.06 (− 0.43, 0.30)	7.29E−01	I2 = 22.5%; Cochrane Q = 72; P = 0.071	No significant outliers		Intercept = 0.005; P = 0.728
Transferrin saturation	Grim	0.24 (− 0.01, 0.49)	6.08E−02	0.18 (− 0.13, 0.49)	2.65E−01	I2 = 0%; Cochrane Q = 38; P = 0.547	No significant outliers		Intercept = -0.001; P = 0.909
Transferrin saturation	Hannum	0.26 (0.01, 0.52)	4.38E−02	0.29 (− 0.02, 0.59)	7.63E−02	I2 = 0%; Cochrane Q = 37; P = 0.626	No significant outliers		Intercept = -0.004; P = 0.700
Transferrin saturation	IE	0.22 (− 0.04, 0.47)	9.78E−02	0.35 (− 0.03, 0.73)	7.80E−02	I2 = 29.4%; Cochrane Q = 57; P = 0.042	No significant outliers		Intercept = -0.009; P = 0.490
Transferrin saturation	Pheno	0.38 (0.03, 0.73)	3.13E−02	0.64 (0.11, 1.17)	2.31E−02	I2 = 45.1%; Cochrane Q = 73; P = 0.001	0.38 (0.11, 0.65)	9.17E−03	Intercept = -0.025; P = 0.183

^a^Heterogeneity in the random effect IVW methods was reported

^b^MR-PRESSO (NbDistribution = 10,000, P < 0.05)

^c^MR-Egger was used to detect pleiotropy. There is no pleiotropy was observed among all analyses (P > 0.05)

CI, confidence interval, MR-PRESSO, Mendelian Randomization Pleiotropy RESidual Sum and Outlier, IE Intrinsic epigenetic

### Causal effects of organic iron content on EAAs

**Fig. 2:**
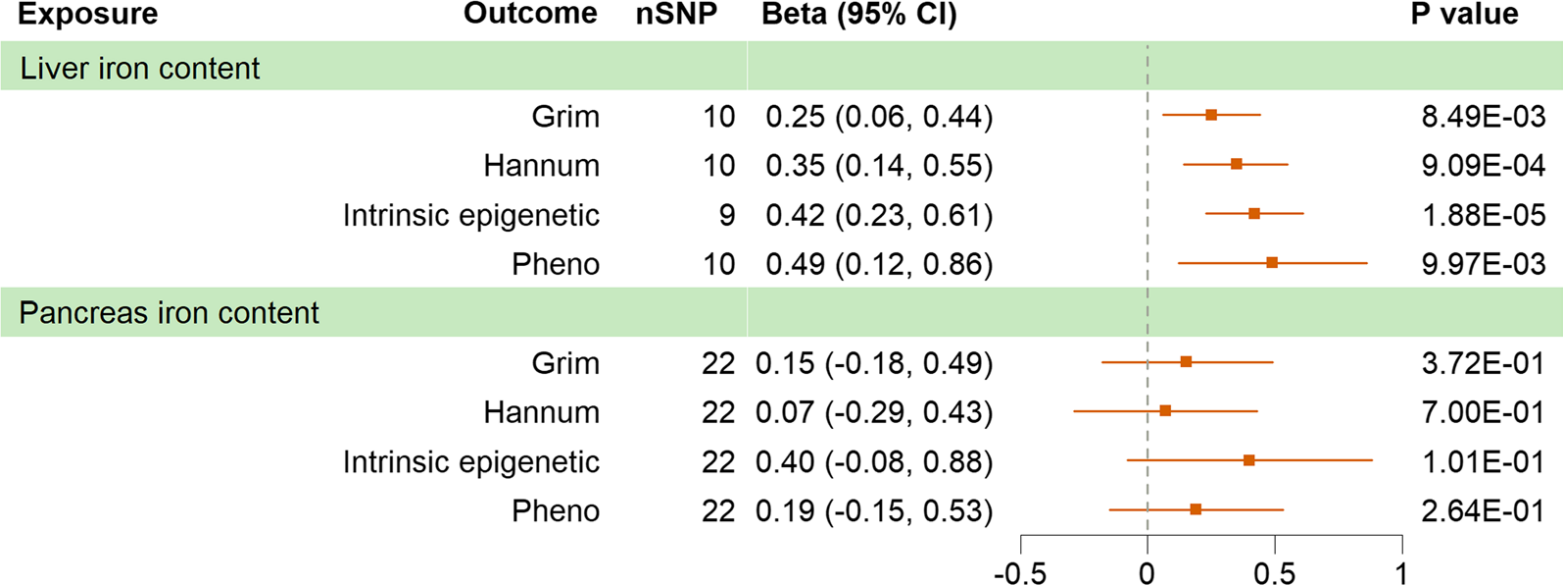
MR analyses of organic iron content to epigenetic aging accelerations. MR, Mendelian randomization; SNP, single nucleotide polymorphism

Genetically predicted liver iron content was associated with increased GrimAA (Beta_IVW_ = 0.25 years per SD increase in liver iron content, P = 8.49E−03), HannumAA (Beta_IVW_ = 0.35, P = 9.09E−04), IEAA (Beta_IVW_ = 0.42, P = 1.88E−05) and PhenoAA (Beta_IVW_ = 0.49, P = 9.97E−03), with no severe heterogeneity detected and no pleiotropy observed. One outliner (rs1799945) was observed by Mendelian Randomization Pleiotropy RESidual Sum and Outlier (MR-PRESSO), removing of which did not influence the result. However, negative results were reported in genetic predisposition to pancreas iron content, despite all analyses returned the same positive direction, as illustrated in Fig. 2 and Table 3. In summary, genetically predicted concentration of iron in liver was associated with acceleration of four epigenetic clocks.

**Table 3:** Sensitivity analyses of organic iron content with epigenetic aging accelerations

Exposure	Outcome	Weighted median		MR-egger regression		Heterogeneity^a^	MR-PRESSO outlier detect^b^		Pleiotropy^c^
		Beta (95% CI)	P value	Beta (95% CI)	P value		Beta (95% CI)	P Value	
Liver iron content	Grim	0.28 (0.05, 0.50)	1.63E−02	0.27 (− 0.03, 0.56)	1.12E−01	I2 = 0%; Cochrane Q = 8; P = 0.538	No significant outliers		Intercept = -0.003; P = 0.892
Liver iron content	Hannum	0.35 (0.13, 0.57)	2.01E−03	0.42 (0.08, 0.76)	3.97E−02	I2 = 21.1%; Cochrane Q = 11; P = 0.249	No significant outliers		Intercept = -0.014; P = 0.598
Liver iron content	IE	0.39 (0.16, 0.62)	8.75E−04	0.32 (0.02, 0.62)	7.22E−02	I2 = 0%; Cochrane Q = 7; P = 0.520	No significant outliers		Intercept = 0.019; P = 0.421
Liver iron content	Pheno	0.39 (0.09, 0.69)	1.19E−02	0.21 (− 0.36, 0.77)	4.95E−01	I2 = 58.5%; Cochrane Q = 22; P = 0.010	0.43 (0.12, 0.74)	2.54E−02	Intercept = 0.052; P = 0.241
Pancreas iron content	Grim	0.11 (− 0.35, 0.57)	6.35E−01	0.57 (− 0.29, 1.43)	2.10E−01	I2 = 4.2%; Cochrane Q = 22; P = 0.404	No significant outliers		Intercept = -0.029; P = 0.316
Pancreas iron content	Hannum	0.08 (− 0.42, 0.58)	7.46E−01	0.50 (− 0.41, 1.41)	2.96E−01	I2 = 9.9%; Cochrane Q = 23; P = 0.328	No significant outliers		Intercept = -0.030; P = 0.328
Pancreas iron content	IE	0.35 (− 0.30, 1.00)	2.87E−01	0.45 (− 0.84, 1.75)	5.00E−01	I2 = 16.4%; Cochrane Q = 25; P = 0.242	No significant outliers		Intercept = -0.004; P = 0.931
Pancreas iron content	Pheno	0.18 (− 0.29, 0.66)	4.48E−01	0.35 (− 0.51, 1.21)	4.35E−01	I2 = 0%; Cochrane Q = 17; P = 0.715	No significant outliers		Intercept = -0.011; P = 0.701

^a^Heterogeneity in the random effect IVW methods was reported

^b^MR-PRESSO (NbDistribution = 10,000, P < 0.05)

^c^MR-Egger was used to detect pleiotropy. There is no pleiotropy observed among all analyses (P > 0.05)

CI, confidence interval, MR-PRESSO, Mendelian Randomization Pleiotropy RESidual Sum and Outlier, IE Intrinsic epigenetic

### Causal effects of EAAs on iron-related traits

To analyze the possible reverse causality, IVW method followed by sensitivity analyses were also performed with EAAs as exposure. The IVW results of EAAs on 2014 datasets, 2021 datasets and organic iron content are pictured in Additional file 1: Figures S2, S3 and S4, with sensitivity analyses on Additional file 1: Tables S1, S2 and S3, respectively. Briefly, none of the genetically predicted EAAs was associated with any of the seven iron-related traits. The sensitivity analyses did not reveal significantly association either. No pleiotropy was observed in every MR-PRESSO test, suggesting reliable results. Collectively, lack of enough evidence to support the causality of EAA on iron-related traits.

## Discussion

In present research, we explored the causal relationship between iron-related biomarkers and epigenetic clocks. In the iron-related traits, genetically predicted serum iron was associated with increasing GrimAA, HannumAA and IEAA. Transferrin carries and transports most of the serum iron to organs and tissues by combining with transferrin receptor of cytomembrane. Serum transferrin was negatively associated with EAAs, despite lack of validation in another GWAS. TIBC is used to describe the maximal capacity of iron transportation in circulation [18]. Transferrin saturation refers to the proportion of transferrin binding with iron, which is normally around 20–40%, derived as serum iron divided by TIBC [3, 19]. Transferrin saturation was associated with all increased EAAs in present study, while TIBC was only significantly associated with IEAA and PhenoAA. Ferritin is responsible for the storage of iron and holds the largest amount of non-functional iron [5]. Serum ferritin from both datasets was associated with PhenoAA, which was supported by results from previous observational study [6]. Liver is rich in iron and plays important role in iron homeostasis. Ferroportin (FPN) is the only cellular exporter of iron, which is regulated by hepcidin synthetized and secreted by liver [20]. Liver is also responsible for recycling iron from aged erythrocytes with Kupffer cells, which export iron to transferrin [3]. In our results, increased liver iron content is associated with all four types of EAAs, which was in agreement with previous results of ferritin levels and ferritin iron saturation in the liver of rats increased with age [21]. However, pancreas iron content displayed no association with EAAs. Although it has been proposed that inflammaging, the aging-driven systemic inflammation, induced the increase of ferritin and was responsible for the iron deficiency and many other diseases [5]. The results did not support the causal relationship of EAA on neither plasmatic nor organic iron content, suggesting other mechanisms involved. Taken together, higher iron concentration in transport and storage were both associated with increased epigenetic age, while lack of concrete evidence to support the causal inference conversely.

To utilize iron in physiological process while avoid its toxicity to the organism, the concentration of iron is strictly modulated in different types of cells, which is typically named as iron homeostasis [3]. Although iron deficiency occurred commonly in the elderly, accumulation of iron in certain organs and tissues was observed, including brain, liver, spleen, kidney, and skeletal muscles, indicating malfunction of iron homeostasis [5, 22]. Iron deposition in senescent cells was observed, which was found associated with increased risk of age-related diseases, including malignancies, neurological disorders and cardiovascular diseases [23].

Several mechanisms have been found to be involved in iron accumulation in senescent cells and its cytotoxic effect. In cells, labile iron pool (LIP) functions as transfer station of imported free iron to stored, and utilized iron. The level of ferritin in senescent cells was elevated, as a result of impaired ferritinophagy, the process of ferritin degradation in lysosome, causing iron sequestration [24]. On the other hand, increased LIP induces the production of reactive oxygen species (ROS), which leads to oxidative stress, lipid peroxidation, and DNA damage, and finally causes damage to cell and promotes cell death [25]. Lipofuscin is also induced by labile iron, the accumulation of which could jeopardize lysosome and promote apoptosis [26]. Ferroptosis, promoted by increased free iron, is involved in malignancies and neurodegeneration [27]. In mitochondria, iron is used to produce heme or iron–sulfur clusters [28]. Overload of iron in mitochondria is related to oxidative stress and malfunction of mitochondria [23].

At the individual level, longevity of organisms could be extended by regulating iron absorption and metabolism. Inhibition of iron absorption has been observed to prolong lifespan in *Drosophila* and *C. elegans* [29, 30]. Reduction of mitochondrial iron in *C. elegans* could also extend the lifespan [31]. *C. elegans*, *Drosophila*, and mice fed with iron-chelating agents demonstrated increased the average lifespan [32]. Deregulation of iron-related genes, including inositol phosphosphingolipid phospholipase C (ISC1), MET18 in yeast, the homologue of methyl-methanesulfonate sensitivity protein 19 (MMS19) in human, frataxin (FXN), have been reported to be associated with shorten lifespan of in vivo models [33–35]. The mammalian target of rapamycin (mTOR) is an important regulator of cell growth and proliferation. Iron activates mTOR, which could be reversed by iron chelators. The activation of mTOR results in iron accumulation via hepcidin, which could be reversed by rapamycin, which was reported to extend the lifespan of mice [36, 37].

In previous study, DNA methylation has been found in connection with iron metabolism. For example, in vitro experiment showed DNA methylation of iron sensing genes modulated the expression of HAMP, who encodes hepcidin [38]. Tibetans with iron overload had a higher ratio of methylation in cytosine-guanine dinucleotide (CpG) compared with normal controls [39]. Iron deficiency was associated with altered DNA methylation in hippocampus of neonate [40]. Serum ferritin of maternal early pregnancy was inversely associated with three CpGs in cord blood [41]. Hemochromatosis (HFE) mutation, which caused hereditary hemochromatosis featured as iron deposition in cells, resulted in attenuated DNA methyltransferase activity and decreased brain methylation in mice [42].

This study is the first MR analysis on iron homeostasis and aging. Epigenetic clock and telomere length have been considered as the most plausible candidates of biological age predictors [12]. In MR study, leukocyte telomere length was commonly used as traits for aging, which could also be influenced by environmental factors [43]. Although telomere length has been widely validated in different kinds of health outcomes, its predictive power was low [12]. Currently, no MR analysis focusing on iron homeostasis and telomere length has been reported. EAA provided more abundant information of impact by both genetic and environmental factors, making it an excellent trait for aging and aging-related outcomes. To make sure external validity of the results, two independent GWAS for serum iron biomarkers were employed, as well as one GWAS for organic iron content. Moreover, only GWAS based on individuals of European ancestry were applied in present study to avoid potential bias due to population stratification.

This study also has several limitations. First, not all four EAAs demonstrated unanimous results in some MR analyses. This is probably due to heterogeneity of EAAs, as they were trained based on different tissues and clinical outcomes. To make results concrete, sensitivity analyses were performed and conclusion would be made only when all analyses exhibited the same direction and no pleiotropy was reported. Second, lack of evidence to support iron content in liver or pancreas to represent iron content of whole body. However, no other GWAS represented body iron content was available. Positive result of liver iron content partially reflected the storage of body, as a compensation to serum ferritin. Third, lack of enough genome-wide significant (P < 5E−08) SNPs when applying pancreas iron content as exposure, which might affect the strength of IVs. No F-statistic of pancreas iron content IVs was less than 20; thus, bias due to weak instrument was unlikely.

Present study demonstrated the potential influence of impaired iron homeostasis, particularly iron overload status, on DNA methylation alteration of human, which represented biological aging and higher mortality risk.

## Conclusions

In a nutshell, our results demonstrated the potential causal relationship of iron overload to accelerate epigenetic clocks. Further researches are required to elucidate the mechanisms and additionally intervene health outcomes via iron homeostasis.

## Methods

**Fig. 3:**
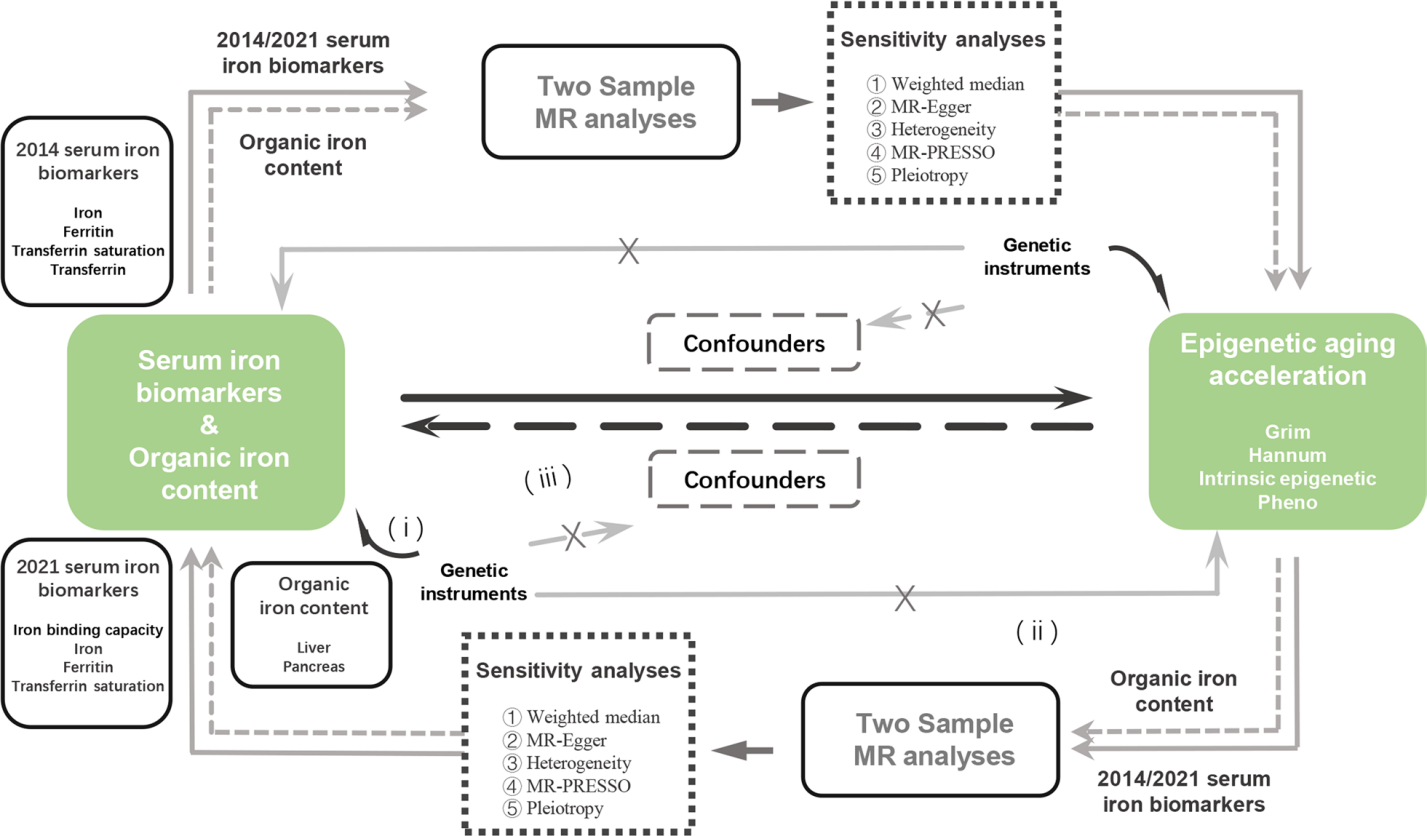
Workflow of present study and basic assumptions of MR analysis. Genetic instruments must be (i) associated with the exposure; (ii) independent of confounders; (iii) independent of the outcome. MR, Mendelian randomization, MR-PRESSO, Mendelian Randomization Pleiotropy RESidual Sum and Outlier

Overview of present study is shown in Fig. 3. The causal relationship of iron homeostasis to EAAs and the reverse causality was explored, with totally eighty of two-sample MR analyses were performed. Sensitivity analyses of each analysis were also carried out.

### GWAS for iron-related traits

A total of five serum iron biomarkers and two organic iron contents were selected to represent the status of iron metabolism. Benyamin et al. reported a meta-GWAS (n = 48,972) for iron homeostasis with four traits, serum level of iron, level of ferritin, level of transferrin and its saturation based on 19 cohorts [44]. Another similar meta-GWAS was constructed by Bell et al., in which individuals of three studies in Ice land, UK and Denmark were enrolled and serum iron (n = 163,551), ferritin (n = 246,139), transferrin saturation (n = 131,471) and TIBC (n = 135,430) were introduced [19]. Liu et al. reported GWAS for iron content in liver (n = 11,069) and pancreas (n = 5,525) by using high-throughput MRI combining deep learning with imaging and clinical data from UK biobank [45].

### GWAS for epigenetic clocks

To measure biological aging, GWAS (n = 34,710) of four types of EAAs reported by McCartney et al. were exploited, which integrated data from 29 studies [16]. To predict chronological age based on the status of DNA methylation, Hannum et al. and Horvath et al. established epigenetic clocks, respectively, now both known as the first generation of epigenetic clocks. The former was built using CpG markers of whole blood cells, which were processed with the Illumina Infinium HumanMethylation450 BeadChip, and the latter applying 51 types of human tissues and cell types analyzed by Illumina Infinium HumanMethylation27 or 450 BeadChip [46, 47]. IEAA, derived from the Horvath clock, was developed to alleviate the influence of varied blood components [16]. To better link epigenetic alterations with age-related outcomes, PhenoAge and GrimAge were developed, which were usually called the second generation of epigenetic clocks. PhenoAge was trained and validated with morbidity and mortality risk (phenotypic age), with DNA methylation sequencing of whole blood from published databases, which exceeded the first generation in predicting mortality [48]. GrimAge was trained on mortality, using DNA methylation data of blood samples from the Framingham heart study (FHS) Offspring Cohort, outperforming the other three in predicting years of time to death [49]. All GWAS included in present study are listed in Table 4.

**Table 4:** Description of GWAS statistic included in present study

Authors	PMID	Traits	Measurement	Data adjustment	Sample size	Sample source	Population	Sex
Benyamin et al. (the 2014 datasets)	25352340	Serum ferritin	Mean, SD	Age, principal component scores and other study specific covariates, for each sex separately, adjustments for each cohort were available in the supplementary of original article	23,986 (Discovery) + 24,986 (Replication)	Genetics of Iron Status Consortium, the Discovery cohorts were composed of 11 cohorts in 9 participating centers, and 8 additional cohorts for the Replication cohorts	European ancestry	Males and Females
	25352340	Serum iron						
	25352340	Serum transferrin						
	25352340	Transferrin Saturation						
Bell et al. (the 2021 datasets)	33536631	Serum ferritin	Rank-based inverse normal transformed SD	Adjusted for age using a generalized additive model. Additionally, for UK cohort the biomarkers were adjusted for menopausal status, ABO blood group, BMI, smoking levels, alcohol levels and iron supplementation status	246,139	Ice land (deCODE genetics), UK (INTERVAL study) and Denmark (Danish Blood Donor Study)	European ancestry	Males and Females
	33536631	Serum iron			163,551			
	33536631	Iron binding capacity			135,430			
	33536631	Transferrin saturation			131,471			
Liu et al.	34128465	Liver iron content	Mean, SD	Age at imaging visit, age squared, sex, imaging center, scan date, scan time, and genotyping batch	11,069	UK Biobank	European ancestry	Males and females
	34128465	Pancreas iron content	Mean, SD		5,525			
McCartney et al.	34187551	GrimAA	Mean, SD	Adjustments for each cohort were available in the supplementary of original article	34,710	29 studies from UK (n = 9), USA (n = 8), Netherlands (n = 3), Finland (n = 2), and Australia (n = 1), Denmark (n = 1), Estonia (n = 1), Germany (n = 1), Italy (n = 1), Sweden (n = 1) and Switzerland (n = 1)	European ancestry	Males and females
	34187551	HannumAA			34,710			
	34187551	Horvath (IEAA)			34,710			
	34187551	PhenoAA			34,710			

SD, standard deviation; GrimAA, GrimAge acceleration; HannumAA, HannumAge acceleration; IEAA, intrinsic epigenetic age acceleration; PhenoAA; PhenoAge acceleration

### Genetic instruments selection criteria

SNPs associated with exposure at a genome-wide significant (P < 5E−08) level were applied in identifying IVs. Restriction was loosened to a threshold of 5E−06 if IVs were less than three in IVW analysis. SNPs in linkage disequilibrium (LD, r^2^ > 0.01, clump window < 10,000 kb) were discriminated and abandoned to keep used SNPs independent based on 1000 Genomes LD reference panel in European ancestry. SNPs with potential weak instrument bias (F-statistic < 10) were removed. SNPs significantly associated with outcome (P < 5E−08) were excluded to avoid violation of MR principles. Elimination of palindromic SNPs was performed using R package “TwoSampleMR” [50]. All the IVs of every MR analysis are listed in Additional file 2: Tables S4–S83.

### MR analyses

To verify the true causality between iron homeostasis and aging, IVW method was applied in this research, as it provided stable causal inference regardless of heterogeneity [51]. Weighted median, MR-Egger regression, heterogeneity test, Cochrane’s Q test and MR-PRESSO were utilized to assess the robustness of IVW. Weighted median model is able to generate consistent estimates, in which more than half of the analytical weights are derived from valid IVs [51]. MR-Egger regression allows pleiotropy in more than half of IVs, while the statistical power is influenced [52]. MR-PRESSO corrects bias due to horizontal pleiotropic outliers [53]. To estimate heterogeneity among SNPs for exposures, Cochrane’s Q test was performed. The conclusion of causal inference was drawn if the same direction results of IVW and all sensitivity analyses were presented, besides no horizontal pleiotropic effect in the intercept test of MR-Egger regression. Finally, to evaluate the strength of IVs for exposures, total F-statistics were calculated [54].

### Statistical analyses

All MR analyses were performed in R 4.2.2 (https://www.R-project.org/). R package “TwoSampleMR” (https://github.com/MRCIEU/TwoSampleMR) [50] “MRPRESSO” (https://github.com/rondolab/MR-PRESSO) [53] were used. A two-sided significance level was set as P value < 0.05 for all statistical testing.

### Supplementary Information

**Additional file 1: Figure S1.** Procedure of IVs selection. **Figure S2.** MR analyses of epigenetic aging accelerations with 2014 datasets serum iron biomarkers. **Figure S3.** MR analyses of epigenetic aging accelerations with 2021 datasets serum iron biomarkers. **Figure S4.** MR analyses of epigenetic aging accelerations with organic iron content. **Table S1.** Sensitivity analyses of epigenetic aging accelerations with serum iron biomarkers in 2014 datasets. **Table S2.** Sensitivity analyses of epigenetic aging accelerations with serum iron biomarkers in 2021 datasets. **Table S3.** Sensitivity analyses of epigenetic aging accelerations with organic iron content.

**Additional file 2: Tables S4–S83.** Instrumental SNPs for serum iron biomarkers, organic iron content and epigenetic aging accelerations in the univariable Mendelian randomization analysis.

## Data Availability

No original data were generated in present study. The datasets supporting the conclusions of this article are available in the Edinburgh DataShare (https://datashare.ed.ac.uk/handle/10283/3645) for GWAS of EAAs [16], GWAS Catalog (https://www.ebi.ac.uk/gwas/publications/25352340) for 2014 datasets [44], deCODE genetics (https://www.decode.com/summarydata/) for 2021 datasets [19], NHGRI-EBI GWAS Catalog for GWAS for organic iron content [45] (accession numbers GCST90016666-GCST90016676, http://ftp.ebi.ac.uk/pub/databases/gwas/summary_statistics/GCST90016001-GCST90017000/GCST90016676/).
