# Supplementary material for: Effects of iron homeostasis on epigenetic age acceleration: a two-sample Mendelian randomization study
Source: Clin Epigenetics. 2023 Oct 7;15:159. doi: 10.1186/s13148-023-01575-w (PMC10559596; doi:10.1186/s13148-023-01575-w)
Supplement: Supplementary file 1 — Additional file 1: Figure S1. Procedure of IVs selection. Figure S2. MR analyses of epigenetic aging accelerations with 2014 datasets serum iron biomarkers. Figure S3. MR analyses of epigenetic aging accelerations with 2021 datasets serum iron biomarkers. Figure S4. MR analyses of epigenetic aging accelerations with organic iron content. Table S1. Sensitivity analyses of epigenetic aging accelerations with serum iron biomarkers in 2014 datasets. Table S2. Sensitivity analyses of epigenetic aging accelerations with serum iron biomarkers in 2021 datasets. Table S3. Sensitivity analyses of epigenetic aging accelerations with organic iron content. [file 13148_2023_1575_MOESM1_ESM.pdf]

SUPPLEMENTAL MATERIAL

Effects of iron homeostasis on epigenetic age acceleration: A Two-sample Mendelian Randomization Study

Zhihao Wang[1†], Yi Liu[2†], Shuxin Zhang[3], Yunbo Yuan[1], Siliang Chen[1], Wenhao Li[1], Mingrong Zuo[1], Yufan Xiang[1], Tengfei Li[1], Yuan Yang[1], Yanhui Liu[1*]

[1] Department of Neurosurgery, West China Hospital, Sichuan University

[2] Department of Thoracic Surgery and Institute of Thoracic Oncology, West China Hospital, Sichuan University

[3] Department of critical care medicine, West China Hospital, Sichuan University

† These authors have contributed equally to this work.

* Corresponding Author: Yanhui Liu, MD, PhD. (liuyh@scu.edu.cn).

Supplementary Figure 1. Procedure of IVs selection.
Supplementary Figure 2. MR analyses of epigenetic aging accelerations with 2014 datasets serum iron biomarkers
Supplementary Figure 3. MR analyses of epigenetic aging accelerations with 2021 datasets serum iron biomarkers
Supplementary Figure 4. MR analyses of epigenetic aging accelerations with organic iron content
Supplementary Table 1. Sensitivity analyses of the epigenetic aging acceleration with the serum iron biomarkers in 2014 datasets.
Supplementary Table 2. Sensitivity analyses of the epigenetic aging acceleration with the serum iron biomarkers in 2021 datasets.
Supplementary Table 3. Sensitivity analyses of the epigenetic aging acceleration with the organic iron content.
Supplementary table 4-83. Instrumental SNPs for serum iron biomarkers, organic iron content and epigenetic aging acceleration in the univariable mendelian randomization analysis.

Supplementary Figure 1. Procedure of IVs selection.

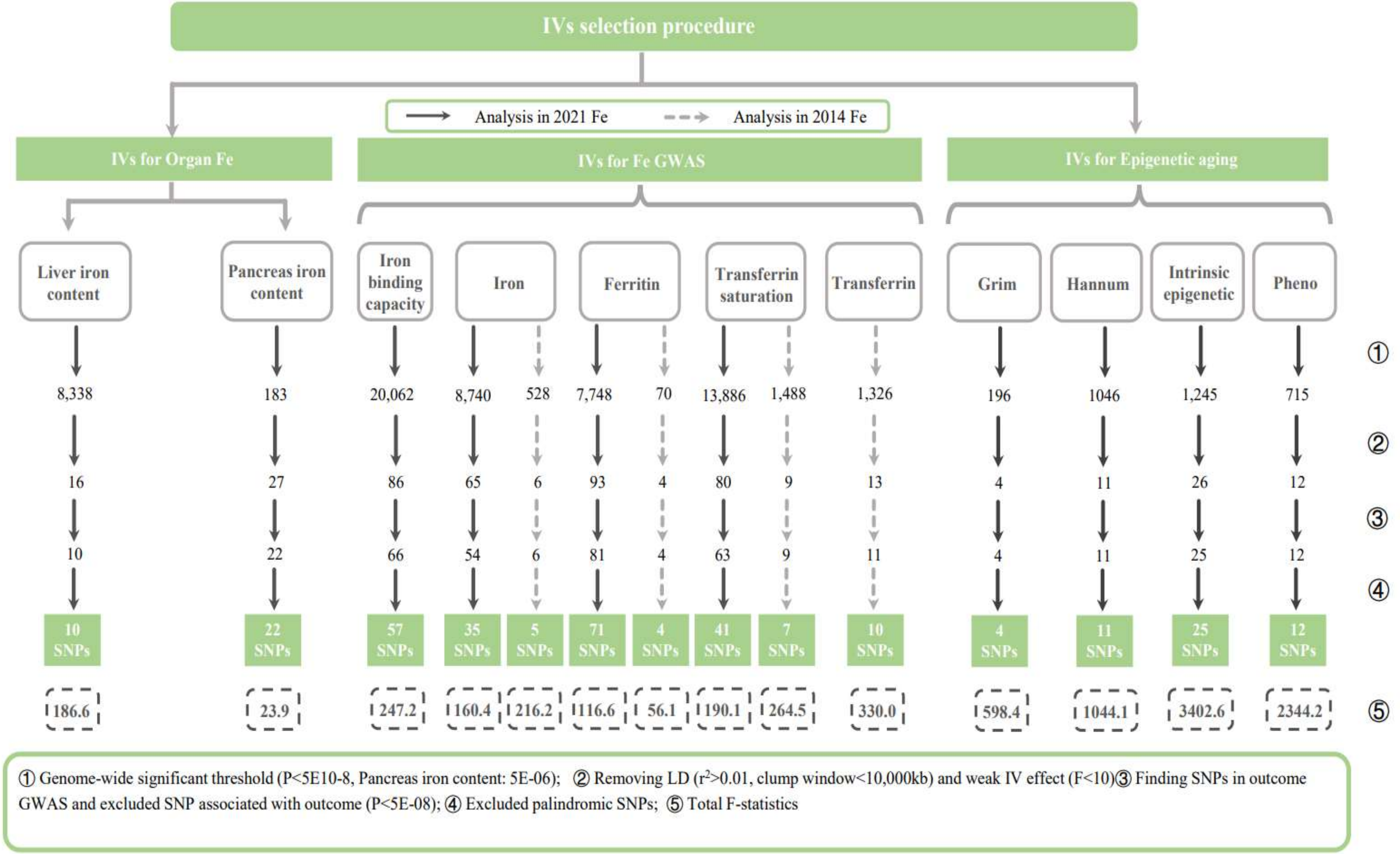

Abbreviations: IVs, instrumental variables; GWAS, genome-wide association study; LD, linkage disequilibrium; SNP, single nucleotide polymorphism.

Supplementary Figure 2. MR analyses of epigenetic aging accelerations with 2014 datasets serum iron biomarkers

Exposure	Outcome	nSNP	Beta (95% CI)	P value
Grim				
	Ferritin	4	0.03 (-0.02, 0.09)	2.38E-01
	Iron	4	0.02 (-0.06, 0.09)	6.58E-01
	Transferrin saturation	4	0.03 (-0.07, 0.12)	5.91E-01
	Transferrin	4	-0.03 (-0.09, 0.04)	4.26E-01
Hannum				
	Ferritin	5	0.00 (-0.04, 0.03)	7.88E-01
	Iron	5	0.00 (-0.04, 0.03)	8.41E-01
	Transferrin saturation	5	0.01 (-0.03, 0.05)	6.16E-01
	Transferrin	5	-0.02 (-0.06, 0.02)	2.86E-01
Intrinsic epigenetic				
	Ferritin	9	0.00 (-0.03, 0.04)	7.85E-01
	Iron	9	0.03 (-0.01, 0.06)	1.32E-01
	Transferrin saturation	9	0.02 (-0.02, 0.06)	2.42E-01
	Transferrin	9	-0.01 (-0.04, 0.03)	7.49E-01
Pheno				
	Ferritin	5	0.01 (-0.01, 0.04)	3.13E-01
	Iron	5	0.02 (-0.02, 0.06)	4.25E-01
	Transferrin saturation	5	0.02 (-0.02, 0.06)	4.28E-01
	Transferrin	5	-0.02 (-0.05, 0.01)	1.71E-01

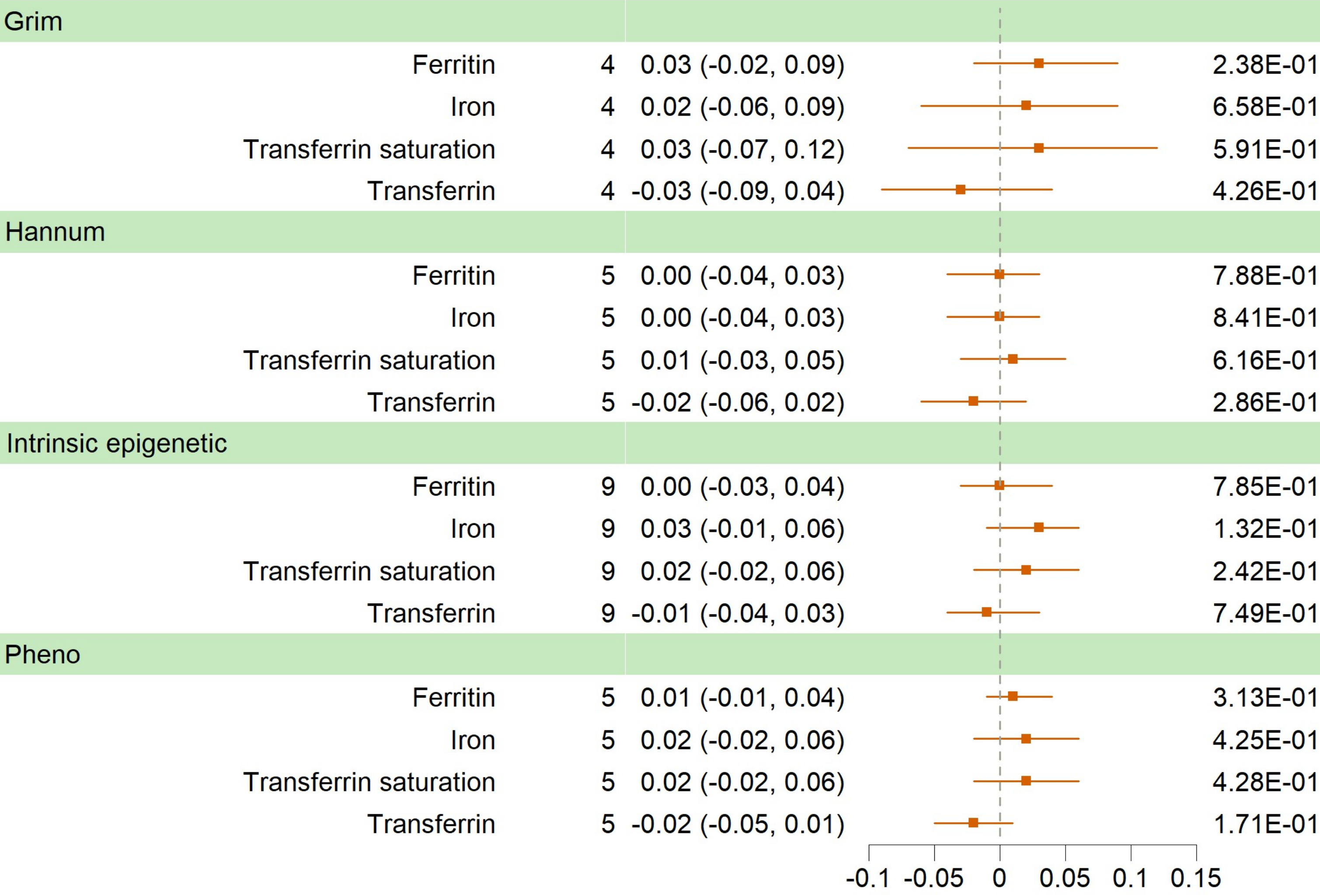

Abbreviations: MR, mendelian randomization; SNP, single nucleotide polymorphism; CI, confidence interval.

Supplementary Figure 3. MR analyses of epigenetic aging accelerations with 2021 datasets serum iron biomarkers

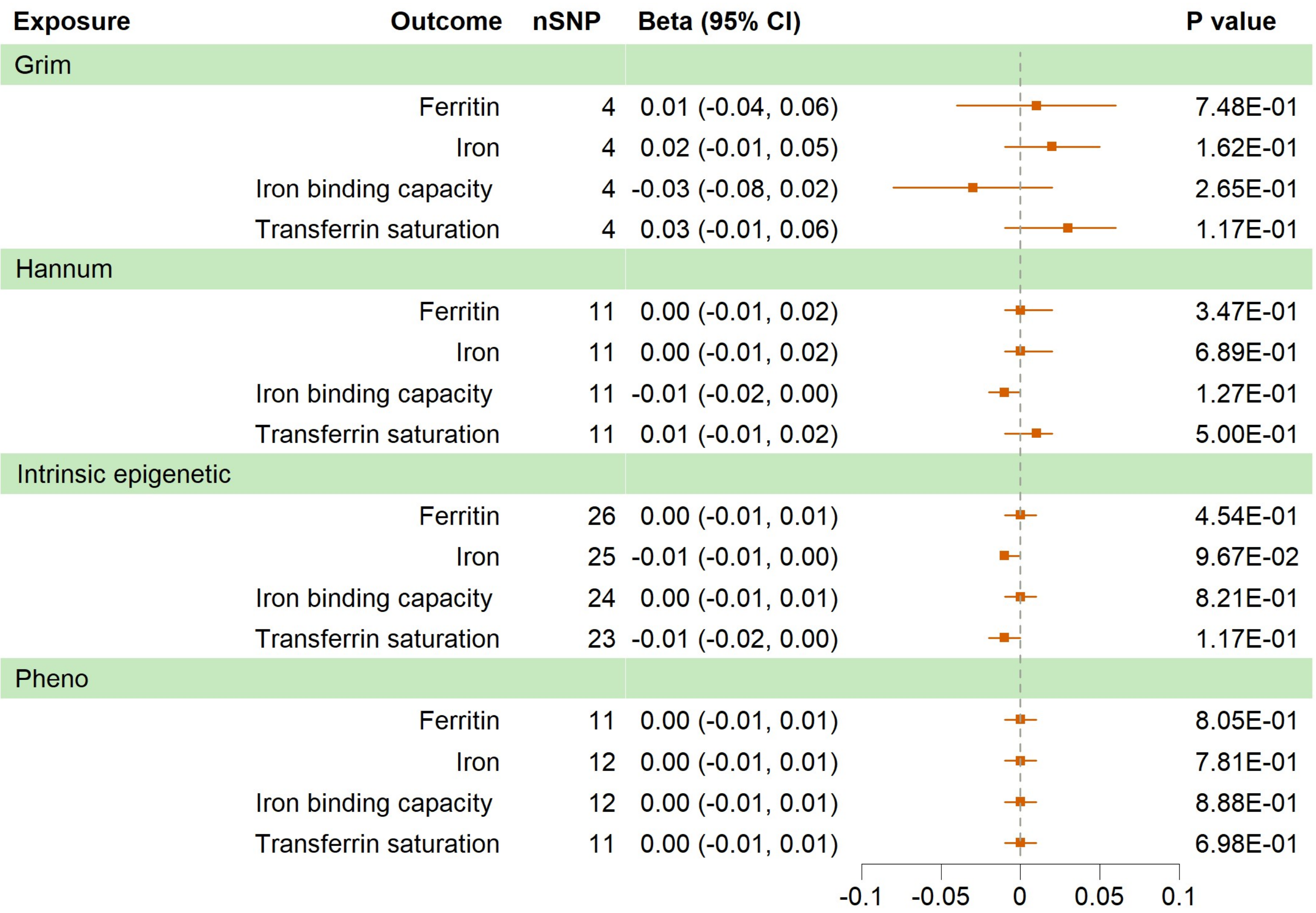

Abbreviations: MR, mendelian randomization; SNP, single nucleotide polymorphism; CI, confidence interval.

Supplementary Figure 4. MR analyses of epigenetic aging accelerations with organic iron content

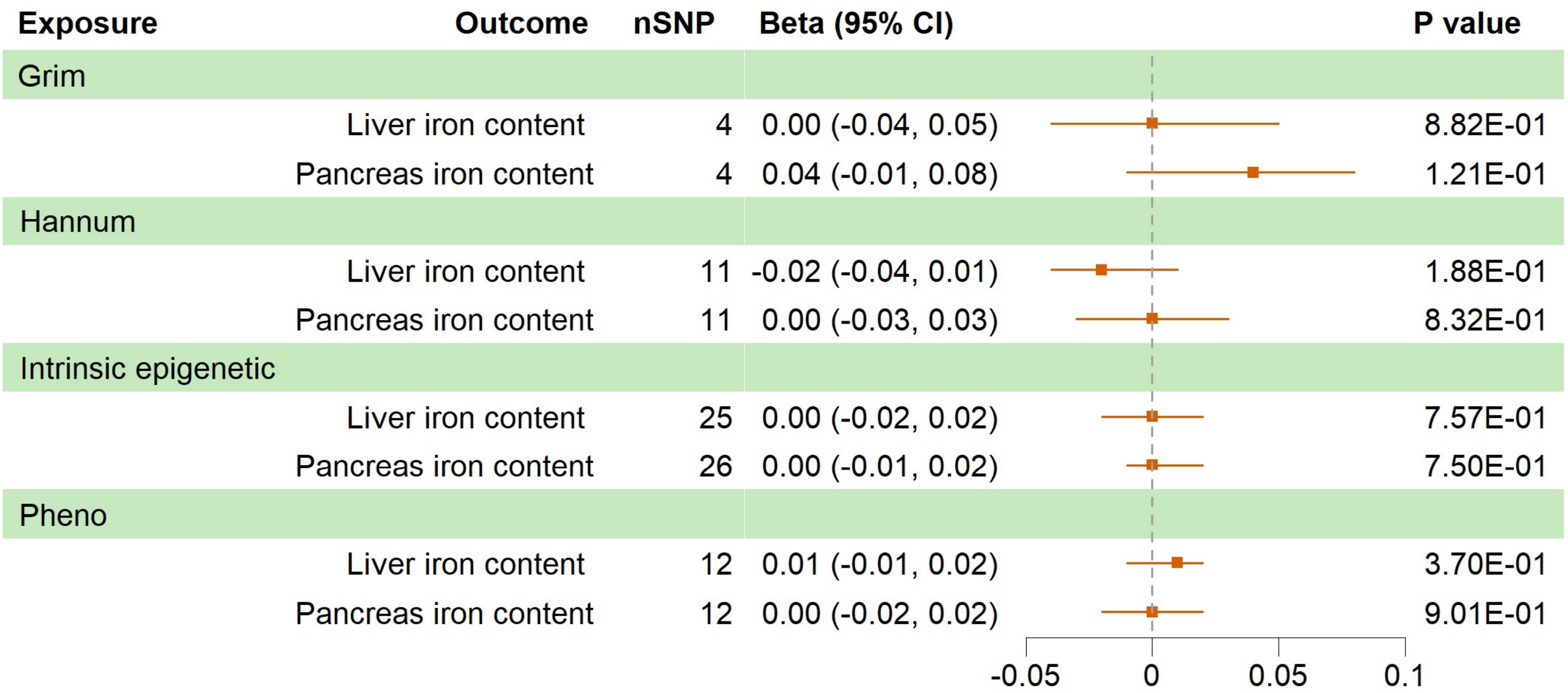

Abbreviations: MR, mendelian randomization; SNP, single nucleotide polymorphism; CI, confidence interval.

Supplementary Table 1. Sensitivity analyses of the epigenetic aging acceleration with the serum iron biomarkers in 2014 datasets.

Exposure	Outcome	Weighted median		MR-Egger regression		Heterogeneity[a]	MR-PRESSO outlier detect[b]		Pleiotropy[c]
		Beta (95% CI)	P Value	Beta (95% CI)	P Value		Beta (95% CI)	P Value	
Grim	Ferritin	0.04 (-0.03, 0.1)	2.56E-01	0.30 (-0.75, 1.35)	6.31E-01	I2 = 14.5%; Cochrane Q = 4; P = 0.32	No significant outliers		Intercept = -0.05; P = 0.667
Grim	Iron	0.01 (-0.06, 0.08)	8.01E-01	0.28 (-1.16, 1.71)	7.42E-01	I2 = 45.3%; Cochrane Q = 5; P = 0.139	No significant outliers		Intercept = -0.048; P = 0.756
Grim	Transferrin Saturation	0.04 (-0.04, 0.11)	3.22E-01	0.05 (-1.82, 1.92)	9.62E-01	I2 = 65.5%; Cochrane Q = 9; P = 0.034	No significant outliers		Intercept = -0.005; P = 0.981
Grim	Transferrin	-0.05 (-0.12, 0.02)	1.72E-01	0.31 (-0.95, 1.58)	6.73E-01	I2 = 30.2%; Cochrane Q = 4; P = 0.231	No significant outliers		Intercept = -0.064; P = 0.648
Hannum	Ferritin	0.00 (-0.04, 0.04)	9.72E-01	-0.01 (-0.17, 0.14)	8.85E-01	I2 = 0%; Cochrane Q = 1; P = 0.955	No significant outliers		Intercept = 0.002; P = 0.929
Hannum	Iron	0.01 (-0.04, 0.05)	7.84E-01	0.08 (-0.08, 0.24)	4.05E-01	I2 = 0%; Cochrane Q = 1; P = 0.840	No significant outliers		Intercept = -0.023; P = 0.374
Hannum	Transferrin Saturation	0.02 (-0.03, 0.07)	4.42E-01	0.08 (-0.08, 0.25)	3.93E-01	I2 = 0%; Cochrane Q = 1; P = 0.881	No significant outliers		Intercept = -0.02; P = 0.433
Hannum	Transferrin	-0.02 (-0.07, 0.03)	4.38E-01	-0.05 (-0.21, 0.12)	6.28E-01	I2 = 0%; Cochrane Q = 1; P = 0.899	No significant outliers		Intercept = 0.007; P = 0.787
IE	Ferritin	0.01 (-0.04, 0.05)	8.07E-01	0.02 (-0.30, 0.34)	8.92E-01	I2 = 16.5%; Cochrane Q =10; P = 0.295	No significant outliers		Intercept = -0.004; P = 0.913
IE	Iron	0.02 (-0.02, 0.07)	3.51E-01	0.20 (-0.13, 0.52)	2.68E-01	I2 = 22%; Cochrane Q = 10; P = 0.248	No significant outliers		Intercept = -0.038; P = 0.332
IE	Transferrin Saturation	0.02 (-0.02, 0.07)	3.70E-01	0.14 (-0.22, 0.49)	4.73E-01	I2 = 26.8%; Cochrane Q =11; P = 0.206	No significant outliers		Intercept = -0.025; P = 0.544
IE	Transferrin	0.00 (-0.05, 0.04)	9.44E-01	0.10 (-0.19, 0.40)	5.18E-01	I2 = 0%; Cochrane Q = 7; P = 0.524	No significant outliers		Intercept = -0.024; P = 0.494
Pheno	Ferritin	0.01 (-0.03, 0.04)	6.81E-01	-0.03 (-0.11, 0.04)	4.55E-01	I2 = 1.1%; Cochrane Q = 4; P = 0.400	No significant outliers		Intercept = 0.021; P = 0.268
Pheno	Iron	0.01 (-0.03, 0.05)	5.81E-01	-0.08 (-0.16, 0.00)	1.53E-01	I2 = 39.9%; Cochrane Q = 7; P = 0.155	No significant outliers		Intercept = 0.041; P = 0.088
Pheno	Transferrin Saturation	0.02 (-0.02, 0.06)	2.62E-01	-0.07 (-0.15, 0.01)	2.04E-01	I2 = 41.1%; Cochrane Q = 7; P = 0.148	No significant outliers		Intercept = 0.036; P = 0.116
Pheno	Transferrin	-0.01 (-0.05, 0.03)	5.37E-01	-0.02 (-0.11, 0.06)	5.95E-01	I2 = 0%; Cochrane Q = 3;	No significant outliers		Intercept = 0.001; P = 0.948

P = 0.629

[a] Heterogeneity in the random effect IVW methods was reported. [b] MR-PRESSO (NbDistribution = 10,000, P < 0.05). [c] MR-Egger was used to detect Pleiotropy. There is no pleiotropy was observed among all analyses (P>0.05). Abbreviation: CI confidence interval, MR-PRESSO Mendelian Randomization Pleiotropy RESidual Sum and Outlier, IE Intrinsic epigenetic.

Supplementary Table 2. Sensitivity analyses of the epigenetic aging acceleration with the serum iron biomarkers in 2021 datasets.

Exposure	Outcome	Weighted median		MR-Egger regression		Heterogeneity[a]	MR-PRESSO outlier detect[b]		Pleiotropy[c]
		Beta (95% CI)	P Value	Beta (95% CI)	P Value		Beta (95% CI)	P Value	
Grim	Ferritin	0.00 (-0.03, 0.02)	8.15E-01	0.24 (-0.80, 1.28)	6.96E-01	I2 = 87.6%; Cochrane Q = 24; P = 0.001	No significant outliers		Intercept = -0.043; P = 0.705
Grim	Iron	0.02 (-0.01, 0.04)	2.30E-01	0.07 (-0.49, 0.63)	8.30E-01	I2 = 37.7%; Cochrane Q = 5; P = 0.186	No significant outliers		Intercept = -0.009; P = 0.877
Grim	Iron binding capacity	-0.03 (-0.06, 0.00)	7.78E-02	-0.54 (-1.23, 0.15)	2.64E-01	I2 = 74.2%; Cochrane Q = 12; P = 0.009	-0.03 (-0.07, 0.01)	0.45	Intercept = 0.095; P = 0.282
Grim	Transferrin saturation	0.02 (-0.01, 0.05)	2.45E-01	0.36 (-0.20, 0.91)	3.34E-01	I2 = 51.2%; Cochrane Q = 6; P = 0.104	No significant outliers		Intercept = -0.061; P = 0.364
Hannum	Ferritin	0.01 (-0.01, 0.02)	2.90E-01	0.00 (-0.03, 0.03)	8.62E-01	I2 = 0%; Cochrane Q = 6; P = 0.791	No significant outliers		Intercept = 0.002; P = 0.601
Hannum	Iron	0.01 (-0.01, 0.03)	2.78E-01	-0.01 (-0.05, 0.04)	7.77E-01	I2 = 45.2%; Cochrane Q = 18; P = 0.051	No significant outliers		Intercept = 0.003; P = 0.66
Hannum	Iron binding capacity	-0.01 (-0.03, 0.01)	2.33E-01	-0.02 (-0.06, 0.02)	4.27E-01	I2 = 0%; Cochrane Q = 5; P = 0.916	No significant outliers		Intercept = 0.002; P = 0.76
Hannum	Transferrin saturation	0.01 (-0.01, 0.03)	3.39E-01	0.00 (-0.05, 0.05)	1.00E+00	I2 = 38.4%; Cochrane Q = 16; P = 0.093	No significant outliers		Intercept = 0.002; P = 0.815
IE	Ferritin	0.00 (-0.01, 0.01)	4.23E-01	0.00 (-0.02, 0.02)	8.72E-01	I2 = 53.5%; Cochrane Q = 54; P = 0.001	0.00 (0.00, 0.00)	1.00	Intercept = 0.001; P = 0.896
IE	Iron	-0.01 (-0.02, 0.00)	9.27E-02	0.00 (-0.02, 0.02)	7.61E-01	I2 = 15.1%; Cochrane Q = 28; P = 0.249	No significant outliers		Intercept = -0.001; P = 0.712
IE	Iron binding capacity	0.00 (-0.02, 0.01)	6.96E-01	-0.01 (-0.03, 0.02)	5.80E-01	I2 = 33%; Cochrane Q = 34; P = 0.061	No significant outliers		Intercept = 0.002; P = 0.612
IE	Transferrin saturation	-0.01 (-0.02, 0.00)	2.02E-01	0.00 (-0.02, 0.03)	8.15E-01	I2 = 47%; Cochrane Q = 42; P = 0.007	-0.02 (-0.02, -0.02)	0.00	Intercept = -0.004; P = 0.345
Pheno	Ferritin	0.00 (-0.01, 0.01)	6.60E-01	0.00 (-0.03, 0.02)	8.32E-01	I2 = 39.2%; Cochrane Q = 16; P = 0.087	No significant outliers		Intercept = 0.001; P = 0.888

Pheno	Iron	0.00 (-0.01, 0.01)	7.78E-01	0.00 (-0.03, 0.03)	9.79E-01	I2 = 37.3%; Cochrane Q = 18; P = 0.093	No significant outliers		Intercept = 0.001; P = 0.898
Pheno	Iron binding capacity	0.00 (-0.01, 0.01)	9.53E-01	0.01 (-0.02, 0.03)	7.01E-01	I2 = 0%; Cochrane Q = 7; P = 0.837	No significant outliers		Intercept = -0.002; P = 0.72
Pheno	Transferrin saturation	-0.01 (-0.02, 0.00)	1.56E-01	0.00 (-0.04, 0.03)	7.53E-01	I2 = 16.3%; Cochrane Q = 12; P = 0.289	No significant outliers		Intercept = 0.001; P = 0.844

[a] Heterogeneity in the random effect IVW methods was reported. [b] MR-PRESSO (NbDistribution = 10,000, P < 0.05). [c] MR-Egger was used to detect Pleiotropy. There is no pleiotropy was observed among all analyses (P>0.05). Abbreviation: CI confidence interval, MR-PRESSO Mendelian Randomization Pleiotropy RESidual Sum and Outlier, IE Intrinsic epigenetic.

Supplementary Table 3. Sensitivity analyses of the epigenetic aging acceleration with the organic iron content.

Exposure	Outcome	Weighted median		MR-Egger regression		Heterogeneity[a]	MR-PRESSO outlier detect[b]		Pleiotropy[c]
		Beta (95% CI)	P Value	Beta (95% CI)	P Value		Beta (95% CI)	P Value	
Grim	Liver iron content	0.02 (-0.03, 0.07)	5.03E-01	-0.26 (-1.05, 0.54)	5.91E-01	I2 = 4.5%; Cochrane Q = 3; P = 0.370	No significant outliers		Intercept = 0.048; P = 0.586
Grim	Pancreas iron content	0.04 (-0.02, 0.09)	1.65E-01	-0.26 (-1.05, 0.52)	5.78E-01	I2 = 0%; Cochrane Q = 1; P = 0.888	No significant outliers		Intercept = 0.056; P = 0.530
Hannum	Liver iron content	-0.02 (-0.05, 0.01)	2.67E-01	0.02 (-0.06, 0.09)	6.80E-01	I2 = 34.8%; Cochrane Q = 15; P = 0.120	No significant outliers		Intercept = -0.010; P = 0.368
Hannum	Pancreas iron content	-0.01 (-0.05, 0.03)	6.06E-01	0.02 (-0.06, 0.11)	6.27E-01	I2 = 31.3%; Cochrane Q = 15; P = 0.149	No significant outliers		Intercept = -0.006; P = 0.656
IE	Liver iron content	0.01 (-0.01, 0.02)	6.06E-01	0.02 (-0.03, 0.07)	4.87E-01	I2 = 50.1%; Cochrane Q = 48; P = 0.002	0.01 (-0.01, 0.03)	2.21E-01	Intercept = -0.004; P = 0.530
IE	Pancreas iron content	0.00 (-0.02, 0.03)	6.64E-01	0.01 (-0.04, 0.05)	7.62E-01	I2 = 22.9%; Cochrane Q = 32; P = 0.146	No significant outliers		Intercept = -0.001; P = 0.841
Pheno	Liver iron content	0.01 (-0.01, 0.03)	2.53E-01	-0.01 (-0.06, 0.04)	6.81E-01	I2 = 6.1%; Cochrane Q = 12; P = 0.385	No significant outliers		Intercept = 0.007; P = 0.449
Pheno	Pancreas iron content	0.00 (-0.03, 0.02)	8.37E-01	-0.02 (-0.07, 0.03)	5.10E-01	I2 = 0%; Cochrane Q = 10; P = 0.564	No significant outliers		Intercept = 0.007; P = 0.454

[a] Heterogeneity in the random effect IVW methods was reported. [b] MR-PRESSO (NbDistribution = 10,000, P < 0.05). [c] MR-Egger was used to detect Pleiotropy. There is no pleiotropy was observed among all analyses (P>0.05). Abbreviation: CI confidence interval, MR-PRESSO Mendelian Randomization Pleiotropy RESidual Sum and Outlier, IE Intrinsic epigenetic.

Supplementary table 4-83: See supplementary file: Supplementary table 4-83.xlsx
